# Towards Sustainable Green Adjuvants for Microbial Pesticides: Recent Progress, Upcoming Challenges, and Future Perspectives

**DOI:** 10.3390/microorganisms11020364

**Published:** 2023-01-31

**Authors:** Fuyong Lin, Yufei Mao, Fan Zhao, Aisha Lawan Idris, Qingqing Liu, Shuangli Zou, Xiong Guan, Tianpei Huang

**Affiliations:** State Key Laboratory of Ecological Pest Control for Fujian and Taiwan Crops & Key Laboratory of Biopesticide and Chemical Biology of Ministry of Education & Biopesticide Research Center, College of Life Sciences & College of Plant Protection, Fujian Agriculture and Forestry University, Fuzhou 350002, China

**Keywords:** surfactant, carrier, protective agent, nutritional adjuvants

## Abstract

Microbial pesticides can be significantly improved by adjuvants. At present, microbial pesticide formulations are mainly wettable powders and suspension concentrations, which are usually produced with adjuvants such as surfactants, carriers, protective agents, and nutritional adjuvants. Surfactants can improve the tension between liquid pesticides and crop surfaces, resulting in stronger permeability and wettability of the formulations. Carriers are inert components of loaded or diluted pesticides, which can control the release of active components at appropriate times. Protective agents are able to help microorganisms to resist in adverse environments. Nutritional adjuvants are used to provide nutrients for microorganisms in microbial pesticides. Most of the adjuvants used in microbial pesticides still refer to those of chemical pesticides. However, some adjuvants may have harmful effects on non-target organisms and ecological environments. Herein, in order to promote research and improvement of microbial pesticides, the types of microbial pesticide formulations were briefly reviewed, and research progress of adjuvants and their applications in microbial pesticides were highlighted, the challenges and the future perspectives towards sustainable green adjuvants of microbial pesticides were also discussed in this review.

## 1. Introduction

In modern agriculture and forestry, pesticides have always played important roles in ensuring agricultural production as a necessity to increase grain production, regulate crop growth, and control plant pests and diseases [1,2]. Following economic globalization, the problems of chemical pesticide residue have attracted the attention of many countries. Many of them have increasingly strict trade standards for pesticide residue in agricultural products. Ideally, pesticides must be lethal to target pests, but not to non-target species, including humans [1,3,4]. Microbial pesticides, mainly produced with naturally occurring bacteria, fungi (including some protozoa and yeasts), and viruses, have been attracting widespread attention due to their advantages in target specificity, environmental safety, efficacy, biodegradability, and applicability in integrated pest management programs [5,6,7].

The United States is the first country in the world to manage pesticide additives. In 1954, the United States FDA implemented the ADI limit. In 1987, according to the policy of “Reducing the potential adverse effects of the use of pesticide products containing toxic inert ingredients”, the United States EPA conducted classified list management on the toxicity and exposure hazards of additives (divided into four categories) [8,9]. The European Union, through the implementation of REACH system, divides pesticide additives into three categories of management, requiring that the limits of 227 substances with potential risks among more than 3200 agricultural chemicals should be marked on the labels, and has clearly stipulated that benzene organic solvents, nonylphenol/octylphenol polyoxyethylene ether, and bovine ester amine surfactants should be restricted or prohibited in pesticide preparations [10]. At present, China has not implemented systematic management of pesticide adjuvants, But a series of regulations have been put in place to regulate some adjuvants [11].

Commercial pesticide preparations are always a mixture of “active ingredients” and “other ingredients” (adjuvants) [12,13]. Biocontrol agents are formulations produced from living organisms or substances produced by them for the control of pests or diseases. Biopesticides are formulations that use living organisms (fungi, bacteria, insect viruses, genetically modified organisms, natural enemies, etc.) or their metabolites (pheromones and auxin, etc.) to kill or inhibit agricultural pests [14,15]. The proportion of bacteria as the main active ingredient of microbial pesticides is the largest, followed by fungi, viruses, and genetically modified microorganisms. *Bacillus thuringiensis* (Bt) bacterial insecticide is the most widely used and most productive microbial insecticide in the world, accounting for 95% of microbial insecticides [16,17,18].

Since the active ingredients of microbial pesticides are mainly microorganisms and their bioactive compounds, their growth and reproduction in the field are affected by environmental factors such as temperature, humidity, and ultra-violet (UV) ray radiation [19]. The microorganisms have strong hydrophobicity and are likely to form large particles in solutions, which make the pesticides difficult to use to solve field application problems such as wettability and suspension [20]. The drawbacks may be overcome by the application of adjuvants to deliver the active ingredients to targets because adjuvants can effectively improve physical and chemical properties of pesticide preparations. There are more than three thousand kinds of pesticide adjuvants in common use. The value of pesticide adjuvants market in 2015 was USD 2.51 billion [19]. To date, adjuvants are developing towards multi-function adjuvants with characteristics that make them labor-saving, low consumption, easy degradation, and low toxicity. They are often applied as wetting agents and emulsifiers in the production of pesticide formulations [21,22].

Green pesticide is harmless to human health, friendly to the environment, ultra-low dosage, high selectivity, and through the green process to produce pesticides [23]. At present, most of the adjuvants used in microbial pesticides still refer to those of chemical pesticides [24]. A green adjuvant is a kind of non-toxic, harmless, and good biodegradable adjuvant added in the process of pesticide production [25]. Biogenic adjuvants are traditionally green, but adjuvants synthesized from fine chemicals are also partially green. Some traditional adjuvants are mostly chemical adjuvants, which have different effects on the active ingredients and the environment. Compared with green adjuvants, it can improve the performance of the preparation and has no impact on the environment. With the development of green agriculture, the safety of pesticides and green pesticide adjuvants is particularly important. [26,27,28]. Herein, in order to promote the research and development of better microbial pesticides, types of microbial pesticide formulations were briefly reviewed, and research progress of adjuvants and their applications in microbial pesticides were highlighted, while the challenges and the future perspectives towards sustainable green adjuvants of microbial pesticides were also discussed.

## 2. Types of Microbial Pesticide Formulations

There are many formulations of microbial pesticides such as wettable powders, suspensions, oil suspensions, water dispersible granules, and suspension seed coatings (Table 1) [29,30,31]. However, the production of microbial pesticides is relatively more complicated than those of chemical pesticides [32,33]. During the preparation process, the microorganisms in the pesticides are more sensitive to external environmental factors such as UV rays, temperature, and humidity [34]. Compared with chemical pesticides, relatively slow action affects the application efficiency of microbial pesticides. The microorganisms, which are mainly bacteria, fungi, or viruses, are insoluble biological particulate matters from 0.5 μm to 1000 μm. They will directly affect the physical properties of the pesticide preparations such as suspension, dispersibility, and wettability [35].

## 3. Classifications and Functions of Adjuvants Used for Microbial Pesticides

The adjuvants used in the production of microbial pesticides are mainly classified into four categories: surfactants, carriers, protective agents, and nutritional adjuvants.

### 3.1. Surfactants

**Figure 1:**
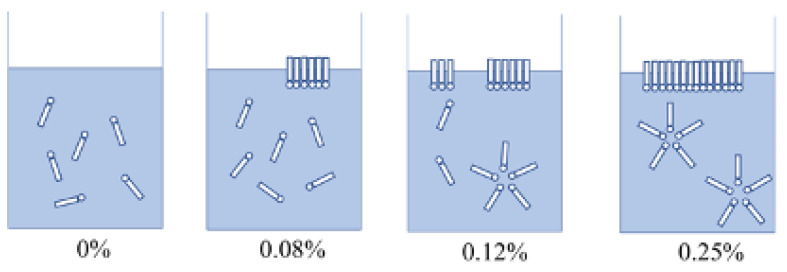
Micelle formation and surface adsorption of surfactants at different concentrations.

Surfactants can improve cost-effectiveness, increase processing efficiency, and save energy and raw materials for the production of microbial pesticides (Figure 1). They play important roles in maintaining long-term physical stability and improving biological functions of the pesticides [36]. About 3.4 million tons of surfactants were produced in 2019, and microbial pesticides usually contain one or more surfactants [37,38].

**Figure 2:**
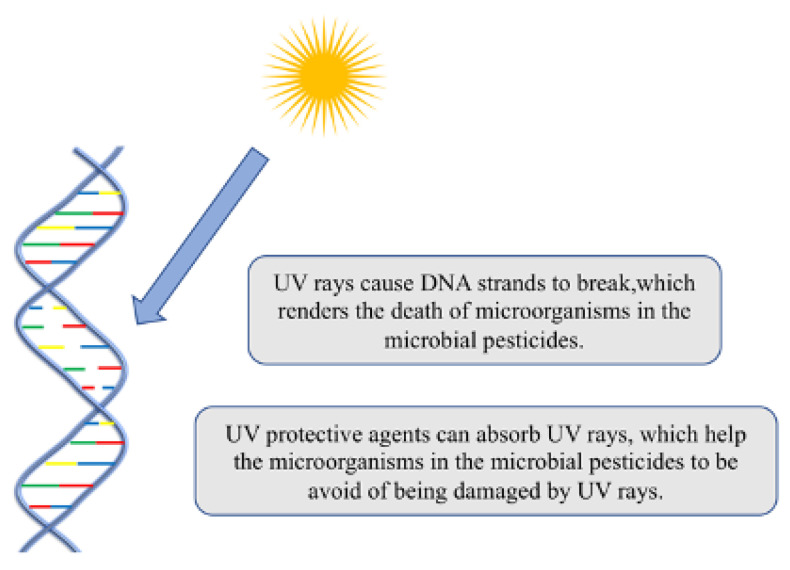
Added protective agents in the microbial pesticides to avoid damage by UV rays.

Surfactants have special molecular structures (amphiphilic structures) consisting of hydrophobic and hydrophilic groups [39,40]. They can be divided into cationic, anionic, nonionic, and amphoteric types by their hydrophilic groups (Table 2 and Figure 2) [41]:

(1) Cationic surfactants: The hydrophilic part is mainly quaternary ammonium ions with strong antibacterial properties [36];
(2) Anionic surfactants: They mainly include ionic sulfonates, sulfates, and carboxylate, most of which contain sodium or calcium. Straight chain alkyl sulfonates occupy the largest market share of all anionic surfactants [42];
(3) Nonionic surfactants: They have polymerized glycol ether and glucose units, which are mainly used as emulsifiers and wetting agents [43,44];
(4) Amphoteric surfactants: Contain both cationic and anionic groups in their structures. They are not only soluble in water, but also highly compatible with other surfactants to form mixed micelles. Their electric charges vary with pH, thereby affecting pesticide properties such as wetting, sedation, and foaming [36].

**Table 2:** Main surfactants used for the production of microbial pesticides.

Types	Main Products	Features	References
Cationic surfactants	Stearyl trimethyl ammonium chloride (STAC) and Hexadecyl trimethyl ammonium bromide (TTAB)	Good water solubility, strong bactericidal power, and adsorption power; cannot be used with anionic surfactants	[45]
Anionic surfactants	Calcium dodecylben-zenesulfonate (ABSCa) and Sodium 2-butyl-1-naphthalenesulfonate (BX)	Good solubility, safety and low toxicity, and strong stability	[46]
Nonionic surfactants	Dibenzyl biphenyl polyoxyethylene ether, Defoamer GP330 and tween-80	Good environmental adaptability, strong stability; cannot be affected by strong electrolytes, acids, and bases	[47]
Amphoteric surfactants	Dodecyl dimethyl betaine (BS-12)	High cost with strong permeability, flocculation, adhesion, resistance reduction, and thickening	[48]

### 3.2. Carriers

The main function of a carrier is to act as a tiny container or diluent for active ingredients of microbial pesticides [20]. The mass percentage of a carrier may exceed that of the active ingredient in the pesticide [49,50]. Carriers such as diatomite, attapulgite, silica, and bentonite with strong adsorption capacities can be used to manufacture high-concentration powders, wettable powders, or granules (Table 3) [51]. Carriers such as talc, pyrophyllite, sepiolite, and clay materials with low or moderate adsorption capacities are generally used as diluents and fillers to produce low-concentration powders (Table 3) [52]. Their pore structures and specific surface areas enable the pesticides to be released into the environments at relatively slow rates [53,54]. Carriers made with biodegradable materials also enable targeted and controlled release of active ingredients from microbial pesticides [55]. For example, chitosan/carbon nanotube nanocomposites were used as the carrier of controlled-release pesticides and prepared, uniformly dispersed carbon nanotube-enhanced Cs films were applied to reduce the harm of pesticides to the environment during the release process [56,57]. Biodiesel may be used as a dilution carrier in liquid pesticide formulations [58]. Some natural plants and synthetic substances are also commonly used as carriers (Table 3) [59].

In the screening process of microbial pesticide carriers, the biocompatibility of carriers and microorganisms in microbial pesticides is the primary concern [67]. Herein, a typical microbial pesticide formulation will select a carrier with good biocompatibility. And the formulation may also rely on wetting and dispersing agents to further improve its wetting and dispersing properties [68]. However, a suitable carrier can confer enough wettability for microbial pesticide formulations without the help of other adjuvants [69]. In fact, carriers greatly affect the wetting, suspending, and dispersing properties of microbial pesticide formulations [35].

### 3.3. Protective Agents

They are mainly divided into the following two categories:

(1) UV protective agents

UV radiation causes passivation, degradation, or damage to microbial pesticides(Figure 2) [70]. UV rays can inhibit the growth of microorganisms, and even cause them to die in severe cases. UV protection agents used for the production of microbial pesticides are mainly divided into UV ray absorbers and anti-oxidative UV protection agents. UV ray absorbers such as locust toxin, optical brighteners, lignosulfonates, and milk can absorb the UV part of sunlight and fluorescent light sources without changing itself [71]. Anti-oxidative UV protection agents such as Rubus oil, hemp seed oil, kojic acid, hydroxykynurenic acid, flavonoids, and lecithin have strong antioxidant effects to help microbial pesticides to avoid being easily oxidized and degraded into other substances that are ineffective against harmful organisms under the irradiation of UV rays. Recently, the development of nanomaterials also provides new ideas for novel UV protective agents [72]. Although UV protective agents such as melanin, berberine, fluorescent whitening agents, and Congo red have significant photoprotective effects on Bt, they are cytotoxic to Bt, and may have a certain impact on the environment [73]. Herein, environmental friendliness should also be considered when UV protective agents are developed.

(2) Other protective agents

Microorganisms in microbial pesticide preparations are easily affected by adverse factors such as temperature, humidity, and oxidation during storage and transportation, which reduce the viable microorganism rate in the formulations and affect their field control effects. Protective agents may also improve the control effect of microbial pesticides in the field and prolong their shelf lives [74]. For example, a protective agent comprised of 8.00% NaCl and 1.00% sodium acetate can increase the spore survival rate of a *Bacillus subtilis* preparation by 22.53% [75]. The addition of protective adjuvants depends on the quality of the biomaterial. In addition to benzoic acid or other chemicals, Bacillus spores can be kept for many years without other protective substances to prevent contamination [76].

### 3.4. Nutritional Adjuvants

During the growth of microorganisms, they need water, inorganic salts, carbon sources, nitrogen sources, and growth factors to grow normally. Nutritional adjuvants can provide nutrients to microorganisms of microbial pesticides to improve their reproductive capacity and promote their proliferation and growth in the field. At present, the research on nutritional adjuvants mainly focuses on the supplementation of carbon and nitrogen sources [77].

## 4. Application of Adjuvants in Microbial Pesticide Formulations

The majority of microbial pesticide formulations are wettable powders and suspension concentrations. Therefore, this section first introduces the applications of adjuvants in them and takes into account other applications.

### 4.1. Application of Adjuvants in Wettable Powders of Microbial Pesticides

A wettable powder refers to a pesticide formulation produced with active ingredients and adjuvants such as carriers, wetting agents, stabilizers, and UV protective agents [78]. The average particle size of a wettable powder is about 44 μm with a ≧ 75% suspension rate and a < 2 min wetting time [79,80]. It can form a stable suspension with good dispersibility after being dissolved and stirred in water and is generally used for spraying in the field [81]. Microbial pesticides, comprised of both microorganisms insoluble in water and organic solvents as the active ingredients, are suitable for processing into wettable powders [82]. Transportation and packaging costs of wettable powders are relatively low. The percentage of active ingredients in the pesticides is higher than that of powders and renders good adhesion to the crops after spraying. However, the powders may not be uniformly dispersed and suspended in the solution during the application, causing problems such as blockage of the nozzle and uneven spraying [83]. Half of registered Bt formulations are wettable powders, which play important roles in the formulations of microbial pesticides [78,84].

Carriers are crucial to wettable powders because the first step of the production is to culture microorganisms to obtain fermentation broths. Substances with strong adsorption properties such as silica, bentonite, attapulgite, and diatomite may be selected as carriers, whose effects on the survival states of microorganisms should also be considered [85]. Biocompatibility is the primary consideration to select a microbial pesticide carrier. At present, the primary evaluation method of biocompatibility is to mix carriers with microorganisms to investigate their survival rates [86]. There are some limitations to this approach. When encountering an adverse external environment, microorganisms will enter dormancy or spore state and survive well, and the carriers cannot show incompatibility with them [87].

Wetting agents such as sodium lauryl sulfate, stretch powder, polyethylene glycol, calcium lignosulfonate, sodium carboxymethyl cellulose, sodium dodecylbenzene sulfonate, sodium tripolyphosphate, lignin, and sodium sulfonate can also be added in the formulations to reduce the interfacial tension between liquid and solid to increase the adhesion of liquid to solid surface [88,89]. In the storage and transportation of microbiological pesticides, the influence of a wetting agent on the water activity of microbiological pesticides should be considered. Under certain water activity conditions, the active microorganism can maintain a relatively good storage state [90].

Stabilizers such as sodium carboxymethyl cellulose, potassium dihydrogen phosphate, calcium carbonate, and methyl cellulose are also key adjuvants for wettable powders [91,92]. Taking *Bacillus* as an example, spores are an important factor to help the bacteria resist adverse environments by protecting the spores from decomposition caused by high temperature [70].

The microorganisms in the pesticides are very sensitive to UV rays in a natural environment. Herein, it is necessary to add UV protective agents to the production of wettable powders [93].

### 4.2. Application of Adjuvants in Suspension Concentrations of Microbial Pesticides

A suspension concentrate, which is formed with active ingredients, carriers, and other adjuvants, is super-pulverized by a sander wet method. Generally, its melting point is higher than 60 °C, which is similar with the characteristics of both emulsifiable concentrates and wettable powders [94]. Suspension concentrates can be mainly classified into water suspension concentrates, oil suspension concentrates, and dry suspension concentrates. They do not use organic substances as solvents and have no risk of inflammability and explosion [82]. They have low toxicities to humans and animals. They are convenient for storage and transportation and can be better sprayed by aircrafts than wettable powders. When applied in the field, they can be mixed with any proportion of water, and easily to adhere to crop surfaces. The production process of a suspension concentrate is relatively simple and environmentally friendly, making it the mainstream formulation for microbial pesticides [95].

The fermentation broth of microorganisms is directly used for the production of suspension concentrates, which are prone to agglomeration, freezing, and precipitation due to the fluidity of the fermentation broth. Herein, adjuvants such as preservatives, dispersants, and stabilizers may be included in the formulations. Preservatives such as potassium sorbate, ethyl p-hydroxybenzoate, and sodium benzoate need to have a good inhibitory effect on mold, yeast, and aerobic bacteria in order to store the suspension concentrations for a long time [96]. The particles of microbial pesticides can automatically aggregate in the solvent, making the surface free energy of the formulations decrease, thereby forming thermodynamically stable systems. A dispersant can make the pesticide particles become smaller, preventing the particles from sedimentation and agglomeration. Dispersants such as calcium lignosulfonate and carboxymethyl cellulose play important roles in maintaining the formulation stability [97]. Stabilizers such as xanthogen glue and magnesium aluminum silicate can control the sizes of the formulation droplets and prevent them from drifting during spraying [98].

### 4.3. Application of Adjuvants in Other Formulations of Microbial Pesticides

Besides the above two formulations, granules, water-dispersible granules, and suspended seed coatings are also often applied in the production of microbial pesticides [99].

Granules are solid and granulated with active ingredients and adjuvants. Their diameter is 300–1700 microns. Their application is convenient, and their absorption and dissolution efficiency are fast. Compared with wettable powders and suspension concentrates, the granules need adjuvants such as binders, colorants, lubricants, and disintegrating agents [75]. Binders, connecting same or different solid materials, are hydrophilic or hydrophobic and indispensable to the formulation. Colorants have functions of warning and classification for microbial pesticides. Lubricants are used to reduce the excessive pressure in the manufacturing process of granules. Disintegrating agents such as NaCl, AlCl_3_, and CaCl_2_ are applied to accelerate the disintegration rates of the granules [100].

Water-dispersible granules are granular formulations that can be rapidly disintegrated and dispersed into a suspension after adding water. Dispersants, binders, and disintegrating agents are indispensable for accelerating their disintegration rate in water [101]. They also need wetting agents, anti-caking agents, and defoamers. The wetting agents are intended to increase the wetting rates of pesticide particles into water and to improve the penetration of water into the granules [102]. Anti-caking agents such as silica gel prevent the formulation from caking by acting as a layer of sliding balls [99].

Suspension seed coatings are flowable and stable uniform suspensions made with active ingredients, adjuvants, and water by wet grinding. The production process is simple and the coating efficiency is high. However, they also have some disadvantages, including aggregation of pesticide particles rendered by long-term storage, and poor coating caused by sedimentation [103]. The formulation needs wetting agents such as alkyl sulfate, lignin sulfur salt, and fatty amine polyoxyethylene ether to reduce the surface tension of solid-liquid interfaces. Xanthan gum can be used to adjust the viscosity of the formulation [104]. Film-forming agents are indispensable to the formulation in that they can bond together and evenly wrap around the outside of the seeds. They are polymer composite materials such as PVA series, PEG macromolecule series, and gum arabic with good air permeability and water permeability. Colorants such as rhododendron in red are also applied to prevent from misuse during storage [105].

## 5. Challenges towards Sustainable Green Adjuvants for Microbial Pesticides

At present, the adjuvants of microbial pesticides are facing many challenges. The author summarizes the following points:

First of all, adjuvants of microbial pesticides are commonly considered to be inert adjuvants without activities. However, some adjuvants may be toxic to non-target organisms and environment [19]. For example, the U.S. Environmental Agency has conducted toxicity analyses on more than 2000 kinds of adjuvants, of which 26% are mutagenic, teratogenic, and carcinogenic (triple effects). They may also have neurotoxicity, endocrine disrupting effects, and cause harmful reproductive damage [59,106].

Secondly, wettable powder formulations of microbial pesticides on the market are very popular with the help of adjuvants [91,107]. However, their particles are relatively coarse, which is not conducive to coverage of crop surfaces, and absorption and utilization of crops.

Finally, the production process of suspension concentrations is relatively simple and cost-effective, but the liquid formulations are not conducive to the storage of microorganisms compared with wettable powders. Drawbacks such as layering, creaming, crystallization, heat storage solidification, room temperature thickening, and short shelf life also need to be solved during the development of suspension concentrations [71,108].

## 6. Future Perspectives

The concept of green pesticides has been gradually accepted by the pesticide industry; consequently, they are actively promoting the use of more environmentally friendly solvents and substances [12,109]. Compared to chemical pesticides, desirable properties of microbial pesticides include target specificity, low environmental persistence, and low non-target biological toxicities [110].

At present, most microbial pesticide adjuvants are very similar to those of chemical pesticides [111]. It is necessary to establish standard administration systems for various adjuvants used for microbial pesticides. That is, all adjuvants of microbial pesticides should be subject to the same risk assessments as that of the active ingredients.

Since the active ingredients of microbial pesticides are microorganisms, the effects of adjuvants on the survival and proliferation of the microorganisms should be considered [59]. That is, biocompatibility between the adjuvants and microorganisms used for microbial pesticides should be determined in storage periods. The effects of adjuvants on the proliferation of microorganisms in crop fields should also be investigated after the application of microbial pesticides.

Focus should also be placed on ensuring adjuvants have stronger adsorption capacity, higher dispersion performance, and better safety in order to enhance biocontrol efficiency of microbial pesticides by improving physical and chemical properties of adjuvants.

Microbial pesticides are key products for the development of sustainable and efficient green agriculture [112]. They will replace highly toxic and highly residual chemical pesticides with the help of sustainable green adjuvants.

## Figures and Tables

**Table 1:** Main formulations of microbial pesticides.

Main Formulations of Microbial Pesticides	Types of Microbial Pesticides	Main Registered Formulations
*Bacillus thuringiensis*	Bacterium	WP, SC, and WG
*Bacillus licheniformis*	Bacterium	SL
*Bacillus cereus*	Bacterium	WP and SC
*Bacillus sphaericus*	Bacterium	SC
*Bacillus subtilis*	Bacterium	WP and FS
*Bacillus amyloliquefaciens*	Bacterium	WP, SC, and WG
*Bacillus velezensis*	Bacterium	WG and TK
*Bacillus firmus*	Bacterium	WP
*Bacillus marine*	Bacterium	WP
*Brevibacterium brevis*	Bacterium	SC
*Paenibacillus polymyxa*	Bacterium	WP and SC
*Pseudomonas fluorescens*	Bacterium	WP and WG
*Beauveria bassiana*	Fungus	WP
*Locust Microsporidia*	Fungus	SC
*Metarhizium anisopliae*	Fungus	WP
*Paecilomyces lilacinus*	Fungus	DP and GR
*Trichoderma*	Fungus	WP and GR
*Verticillium pachyspora*	Fungus	WP
*Autographa californica* nuclear polyhedrosis virus	Virus	SE
*Ectropis obliqua* nuclear polyhedrosis virus	Virus	TK
*Helicoverpa armigera* nuclear polyhedrosis virus	Virus	WP and SC
*Mamestra brassicae* nuclear polyhedrosis virus	Virus	WP, SC, GR, and TK
*Plutella xylostella* granulosa virus	Virus	WP
*Pierisrapae granulosis*	Virus	TK
*Spodoptera exigua* nuclear polyhedrosis virus	Virus	SC, WG, and TK
*Spodoptera litura* nuclear polyhedrosis virus	Virus	WP, SC, and WG

Wettable powder(WP), suspension concentration(SC), dispersible granules(WG), soluble liquid(SL), seed treatment(FS), granules(GR), powder(DP), technical concentration(TK).

**Table 3:** Carriers commonly used for the production of microbial pesticides.

Types of Carriers	Features	References
Bentonite	Its main component is montmorillonite, which has strong adsorption and large specific surface area. After absorbing a large number of water molecules, it expands and splits into extremely fine particles.	[60]
Diatomite	Its main component is SiO2, which harbors many micropores and has a capacity of low relative density, high porosity, and strong adsorption. It is widely used in the manufacture of high-concentration powder carriers.	[61]
Attapulgite clay	Its main component is attapulgite, which has a capacity of strong adsorption and a property of large specific surface area and unique thickening. It is widely used in the manufacture of high-concentration powder carriers and granule substrates, as well as thickeners for suspensions.	[57]
Kaolin	Its main component is kaolinite, which has a relatively compact structure, a small specific surface area, and an adsorption capacity. It is often used as a carrier for low-concentration powders, and the price is relatively cheap.	[57]
Zeolite	Active ingredients in forms of porous hydrous aluminosilicate crystals are highly adsorbable to certain polar molecules such as zeolite and then slowly released. It is often used as a carrier for sustained-release granules.	[62]
Sepiolite	Its main component is magnesium-rich fibrous clay minerals with large porosity and specific surface area. It can absorb liquid and low-melting pesticides. Because of its light weight, it can float on the water surface.	[63]
Synthetic vectors	Most of them are made with light calcium carbonate and white carbon black, which have strong specific surface area and adsorption capacity, and can be used as a carrier for high concentration powders.	[64]
Plant vectors	Most of them are made with bagasse, corn bagasse, chaff powder, tobacco powder, and walnut shell powder. Plant-based carriers are rarely used at present. Some of them have special properties such as absorbing ultraviolet (UV) rays.	[65]
Nanostructured Lipid Carriers	They have excellent permeability, retention, targeting, stability, and can reduce or eliminate the side effects of active ingredients, with good slow release and controlled release properties.	[66]

## Data Availability

Not applicable.
